# Risk-Adjusting Mortality in the Nationwide Veterans Affairs Healthcare System

**DOI:** 10.1007/s11606-021-07377-1

**Published:** 2022-01-13

**Authors:** Hallie C Prescott, Rajendra P Kadel, Julie R Eyman, Ron Freyberg, Matthew Quarrick, David Brewer, Rachael Hasselbeck

**Affiliations:** 1 VA Center for Clinical Management Research, Ann Arbor, MI USA; 2 University of Michigan, Department of Medicine, Ann Arbor, MI USA; 3 VA Inpatient Evaluation Center, Department of Veterans Affairs, Veterans Health Administration, 810 Vermont Ave. NW Room 668, Washington, DC 20420 USA; 4 VA Center for Strategic Analytics and Reporting, Department of Veterans Affairs, Veterans Health Administration, 810 Vermont Ave. NW Room 668, Washington, DC 20420 USA

**Keywords:** hospital mortality, risk adjustment, logistic models

## Abstract

**Background:**

The US Veterans Affairs (VA) healthcare system began reporting risk-adjusted mortality for intensive care (ICU) admissions in 2005. However, while the VA’s mortality model has been updated and adapted for risk-adjustment of all inpatient hospitalizations, recent model performance has not been published. We sought to assess the current performance of VA’s 4 standardized mortality models: acute care 30-day mortality (acute care SMR-30); ICU 30-day mortality (ICU SMR-30); acute care in-hospital mortality (acute care SMR); and ICU in-hospital mortality (ICU SMR).

**Methods:**

Retrospective cohort study with split derivation and validation samples. Standardized mortality models were fit using derivation data, with coefficients applied to the validation sample. Nationwide VA hospitalizations that met model inclusion criteria during fiscal years 2017–2018(derivation) and 2019 (validation) were included. Model performance was evaluated using c-statistics to assess discrimination and comparison of observed versus predicted deaths to assess calibration.

**Results:**

Among 1,143,351 hospitalizations eligible for the acute care SMR-30 during 2017–2019, in-hospital mortality was 1.8%, and 30-day mortality was 4.3%. C-statistics for the SMR models in validation data were 0.870 (acute care SMR-30); 0.864 (ICU SMR-30); 0.914 (acute care SMR); and 0.887 (ICU SMR). There were 16,036 deaths (4.29% mortality) in the SMR-30 validation cohort versus 17,458 predicted deaths (4.67%), reflecting 0.38% over-prediction. Across deciles of predicted risk, the absolute difference in observed versus predicted percent mortality was a mean of 0.38%, with a maximum error of 1.81% seen in the highest-risk decile.

**Conclusions and Relevance:**

The VA’s SMR models, which incorporate patient physiology on presentation, are highly predictive and demonstrate good calibration both overall and across risk deciles. The current SMR models perform similarly to the initial ICU SMR model, indicating appropriate adaption and re-calibration.

**Supplementary Information:**

The online version contains supplementary material available at 10.1007/s11606-021-07377-1.

## INTRODUCTION

The United States Veterans Affairs (VA) healthcare system is the nation’s largest integrated healthcare delivery system, with approximately 550,000 acute care hospitalizations annually to 140 acute care hospitals.^1^ Starting in 2005, the VA began to measure and report risk-adjusted mortality for patients admitted to intensive care units (ICUs) for the purpose of performance assessment and improvement.^2,3^ Tracking risk-adjusted mortality is helpful for evaluating changes over time, evaluating changes in response to specific policies or performance improvement initiatives, and identifying hospitals with greater-than-predicted mortality for further review.

The VA’s risk-adjustment model includes data on patients’ demographics, chronic health conditions, admitting diagnosis, and physiology within the first 24 h of admission, similar to the Acute Physiology and Chronic Health Evaluation (APACHE^4^) measure^3^. The development, validation, and first re-calibration of the ICU mortality model were published previously.^2,3^ Over the past 15 years, however, the mortality model has been adapted for risk adjustment of all inpatient hospitalizations, updated to incorporate additional variables, and re-calibrated annually to account for temporal changes in diagnosis, coding, medical management, and outcomes. Periodic re-fitting of risk-adjustment models is necessary to prevent model performance from degrading over time.^5,6^ Consistent with Centers for Medicare and Medicaid Services, the VA mortality models are re-calibrated annually.

Given the expansion and revision of VA mortality models over time since publication of the original VA ICU mortality model, we sought to evaluate the performance of VA’s mortality models in a recent sample of hospitalizations. Specifically, we tested the models’ discrimination, assessed the models’ calibration, and examined the stability of model performance across quarters. While we examined all four mortality models in operational use, we focused on the acute care 30-day mortality model because it is the most comprehensive (includes both ward and ICU patients, and captures both in-hospital and post-discharge mortality) and therefore the most important mortality model for overall performance assessment.

## METHODS

### Setting

The VA healthcare system is an integrated healthcare delivery system that provides comprehensive healthcare to Veterans. The VA was among the first healthcare delivery systems to have a universal electronic health record, and to measure and report risk-adjusted mortality.^1^

### Mortality Models

As part of routine performance assessment, the VA measures and reports four standardized mortality ratios (SMRs) for each VA hospital on a quarterly basis: (1) acute care 30-day mortality (acute care SMR-30); (2) ICU 30-day mortality (ICU SMR-30); (3) acute care in-hospital mortality (acute care SMR); and (4) ICU in-hospital mortality (ICU SMR). The mortality models are each developed on a rolling 2-year look-back of VA hospitalizations, then applied to the current fiscal year. The inclusion criteria, definitions, and key differences of each SMR model are presented in Appendix 1 and Supplemental Table 1. A summary of key changes to the models since their last description is presented in Supplemental Table 2.

For the acute care models, predicted mortality is estimated using a logistic regression model that includes the following predictors: age, admitting diagnosis category, major surgical procedure category, 29 comorbid conditions, physiologic variables (sodium, BUN, creatinine, glucose, albumin, bilirubin, white blood cell count, and hematocrit), immunosuppressant status, ICU stay during hospitalization, medical or surgical diagnosis-related grouping (DRG), source of admission (e.g., inter-hospital transfer, nursing facility), and marital status. For physiologic variables, the most deranged value within a specified time frame is included in this statistical model. For non-operative patients, this time frame is between 24 h prior to hospital admission and 24 h after hospital admission. For operative patients, this time frame is between 14 days prior to hospital admission and 24 h after hospital admission. Normal values are imputed for missing physiologic variables, as is conventional for risk adjustment.^7^ The admitting diagnosis category assigns all possible admitting diagnoses to one of 51 mutually exclusive groupings, which were consolidated from the Healthcare Cost and Utilization Project’s Clinical Classification Software categories^8^ based on clinical similarity and on the observed mortality rate. Similarly, the major surgical procedure category includes 24 mutually exclusive groupings based on major surgical procedures within 24 h of presentation. Comorbid conditions are identified from diagnostic codes during hospitalization, using the methods of Elixhauser et al., adapted for ICD-10 coding.^9,10^ Immunosuppressant status is defined based on use of immunosuppressive medications in the 90 days prior to hospitalization.^11^ The ICU models include additional physiologic variables (PaO_2_, PaCO_2_, and pH) as well as hospital length of stay prior to ICU admission.

### Model Performance

For this study, the SMR models were developed using hospitalizations from fiscal years (FY) 2017–2018, and model performance was assessed using hospitalizations in FY 2019. Thus, the study examines a recent, but pre-pandemic, cohort of hospitalizations. We evaluated model performance using c-statistics to assess discrimination and comparison of observed vs predicted deaths by decile of predicted risk to assess calibration (i.e., the agreement between observed outcomes and predictions).^6,12–14^ The c-statistic is a measure of goodness of fit for binary outcomes of a logistic regression model, and tells the probability that a randomly selected hospitalization that had mortality had a higher predicted risk than a randomly selected hospitalization that did not experience mortality.^15,16^ Additionally, we report Hosmer-LemeshowGoodness-of-Fitchi-square and Brier scores (to harmonize with a prior study of the VA’s mortality model^3^), as well as mean and maximum difference in observed versus predicted percent mortality across deciles of risk to summarize the model calibration^14^. We considered model discrimination to be strong when c-statistic was >0.8, consistent with standard practice.^15,16^ We are not aware of any generally accepted threshold for grading model calibration,^12,13,17^ but considered overall and mean calibration errors of <1.0% to reflect good model calibration.

We assessed model performance in the derivation cohort, the validation cohort, and by quarter for the validation cohort. For the ICU models, we also assessed model performance by level of intensive care, as defined by availability of subspecialty services.^18^ Finally, for the acute care SMR-30 model, we evaluated c-statistics in a series of nested models to understand the incremental impact of administrative and clinical data on model discrimination.

Data management and analysis were completed in SAS Enterprise Guide 8.3 (SAS Institute Inc., Cary, NC). Figures were produced in R. This study was approved by the Ann Arbor VA Institutional Review Board with a waiver of informed consent.

## RESULTS

### Cohort Characteristics

Among 1,996,645 inpatient stays during fiscal years 2017–2019, there were 1,143,351 acute care hospitalizations meeting criteria for the Acute Care SMR-30. Of 1,996,645 inpatient stays, 673,813 (33.7%) were excluded due to a non-acute care treating specialty (e.g., nursing, psychiatry, and rehabilitation care), 114,068 (5.7%) because they occurred within 30 days of a prior hospitalization, 1,280 because they involved specialized treatments (organ transplantation of left ventricular assist device) (0.1%), 415 patients who died within 4 h of arrival (0.02%), and 20,011 with hospice care during the calendar day of admission or the preceding year (1.0%). Study flow diagrams showing the application of model exclusions for each SMR model are presented in Supplemental Tables 3 and 4, while Supplemental Table 5 shows the number of unique patients in the acute care and ICU SMR 30 models.

**Figure 1:**
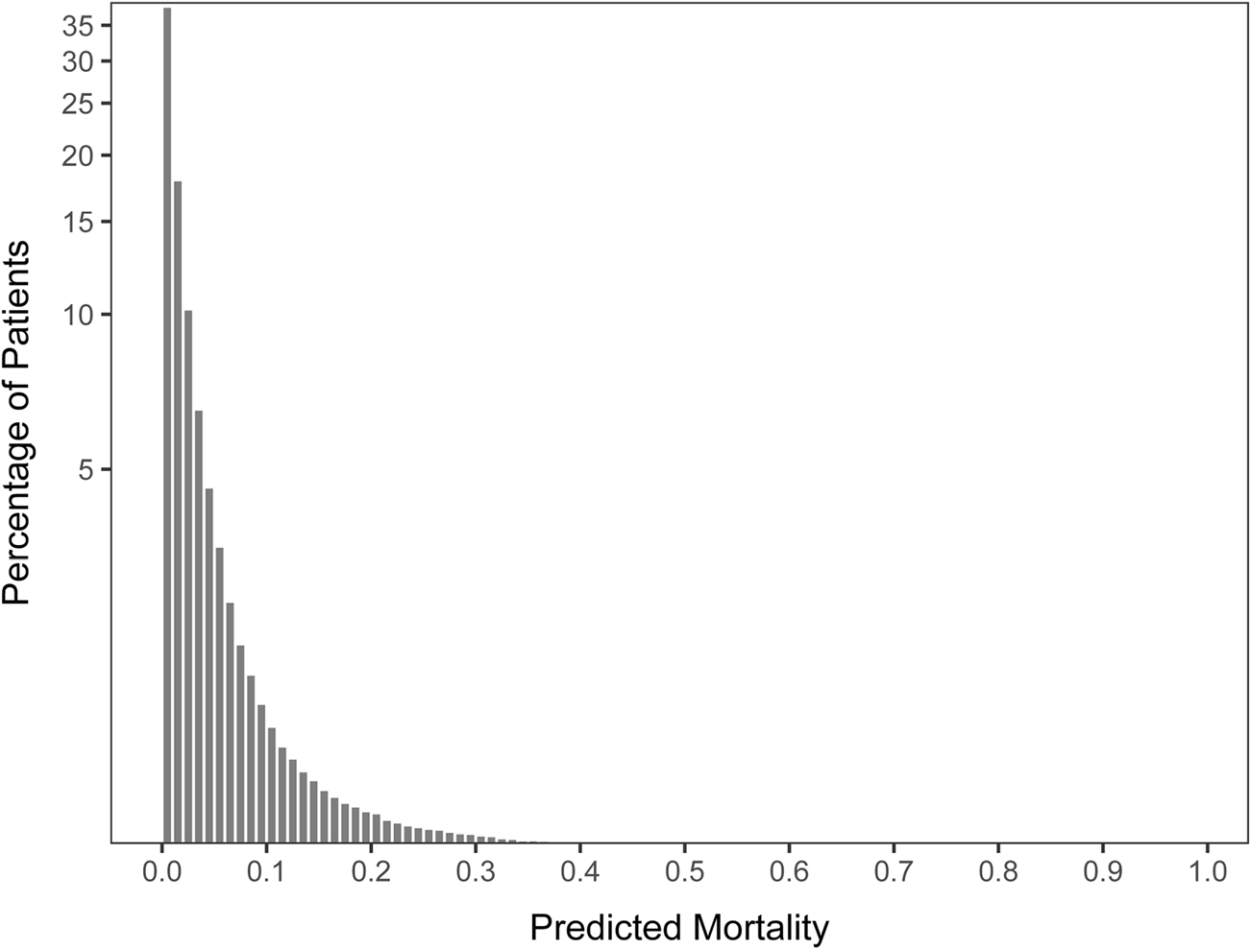
**Histogram showing the distribution of predicted risk of 30-day mortality for the Acute Care SMR-30 Validation Cohort. Predicted 30-day mortality for the SMR-30 derivation cohort was a median of 1.5%, mean 4.3%, IQR 0.5%, 4.1%. For the validation cohort, median 1.6%, mean 4.7%, IQR 0.6%, 4.4%. The*y*-axis uses a pseudo-log transformation with a smooth transition to linear scale around 0.**

Acute care SMR-30 cohort characteristics and outcomes are presented in Table 1. Hospitalizations in the SMR-30 model were median age 68 years (IQR 61–74), 94.4% male, and 70.8% White, with a median of 3 comorbid conditions (IQR 2, 5). The majority of hospitalizations were admitted via the emergency department or directly, while 15.1% were admitted from the operating room, 2.4% were transferred in from another hospital, and 1.9% were admitted from nursing facilities. The most common admission diagnosis categories were musculoskeletal injuries (7.5%), congestive heart failure (5.7%), non-sepsis/non-pneumonia infections (5.6%), neurological diseases (5.2%), and sepsis (4.5%). In-hospital mortality was 1.8%, and 30-day mortality was 4.3%. Patient characteristics were similar between the derivation (FY 2017-2018) and validation (FY 2019) cohorts. For the acute care SMR-30 validation cohort, predicted risk of 30-day mortality was median 1.6% (IQR 0.6%, 4.4%), mean 4.7% (Fig. 1).

**Table 1:** Patients and Hospitalization Characteristics for Derivation, Validation, and Full Cohort for the Acute Care SMR-30 Model

	Total cohort FY 2017–2019	Derivation cohort FY 2017–2018	Validation cohort FY 2019
Hospitalizations, *N*	1,143,351	769,924	373,727
Unique patients, *N*	702,866	512,868	198,998
Male, % of hospitalizations	94.4%	94.5%	94.2%
Self-reported race, % of hospitalizations			
White	70.8%	71.1%	70.1%
Black or African American	21.5%	21.3%	22.1%
Other, unknown, or not reported	7.7%	7.6%	7.8%
Age, median (IQR)	68 (61,74)	68 (61,74)	68 (61,75)
Number of comorbid conditions, median (IQR)	3 (2,5)	2 (2,4)	3 (2,5)
Select comorbid conditions, % of hospitalizations			
Congestive heart failure	12.7%	12.2%	13.7%
Chronic pulmonary disease	23.9%	23.6%	24.7%
Liver disease	7.7%	7.6%	8.0%
Metastatic cancer	2.4%	2.4%	2.6%
Immunosuppressed status (indicator in SMR models)	22.5%	22.2%	23.1%
Admission Source			
Other hospital (VA or non-VA)	2.4%	2.3%	2.5%
Nursing home (VA or non-VA)	1.9%	1.8%	2.0%
Operating Room	15.1%	15.3%	14.7%
Other (Emergency Department, Direct Admission)	80.6%	80.6%	80.8%
Admitting diagnosis category*, % of hospitalizations			
Musculoskeletal (besides hip fracture)	7.5%	7.5%	7.5%
Congestive heart failure	5.7%	5.7%	5.9%
Infections (besides sepsis or pneumonia)	5.6%	5.7%	5.3%
Neurologic diseases	5.2%	5.2%	5.1%
Sepsis	4.5%	4.2%	5.1%
Substance-related disorders	4.3%	4.2%	4.4%
Chronic obstructive pulmonary disease	4.0%	4.1%	3.9%
Cardiac dysrhythmias	3.7%	3.6%	3.7%
Other gastrointestinal disease	3.7%	3.8%	3.7%
Complications of surgical/medical care	3.5%	3.5%	3.6%
All other diagnosis categories	52.3%	52.5%	51.8%
DRG grouping, % of hospitalizations			
Medical	77.4%	76.8%	78.5%
Surgical	21.6%	21.7%	21.3%
Major surgical procedure within 24 h, %	15.5%	15.7%	15.1%
Length of hospitalization in days, median (IQR)	2 (1,4)	3 (1,4)	2 (1,4)
ICU admission, % of hospitalizations			
Directly admitted directly to ICU	14.4%	14.4%	14.2%
Ever admitted to ICU	18.0%	18.1%	17.8%
Mortality, % of hospitalizations			
In-hospital	1.8%	1.7%	1.8%
30-day	4.3%	4.3%	4.3%

*Hospitalizations were each assigned to one of 51 mutually exclusive diagnosis categories based on their admitting diagnosis. These diagnosis categories each include one or more clinical classification software^8^(CCS) diagnosis categories. The consolidation of CCS categories into admission diagnosis categories was informed by clinical rationale, as well as by the observed mortality rates for CCS categories. For example, the musculoskeletal category is relatively large because it combines several low-risk CCS categories including diagnoses related to back, knee, facial, and extremity injury or illness, but hip fracture is kept as a separate category due to its higher associated mortality

### Model Performance

In total, across the 4 SMR models, we assessed model performance for 24 different scenarios in the validation data, as defined by the model of interest, time-period of interest, and (for the ICU models only) level of intensive care available (Table 2). Overall, the c-statistic ranged from 0.848 to 0.918 across the 24 scenarios, indicating that model performance was consistently strong. When examining nested models for the SMR-30 model, c-statistic was 0.840 in a basic administrative model, 0.853 in an enhanced administrative model, and 0.870 in the full model—showing the added benefit of including physiological data (Supplemental Table 6).

**Table 2:** Performance of the SMR Models in Derivation and Validation Cohorts

Model and Cohort	*N*	Mortality, *N* (%)	C stat	H-L GOF chi-square	Brier’s score
Acute Care SMR-30 derivation	769710	33180 (4.31)	0.871	187.7	0.035
Acute Care SMR-30 validation, full year	373791	16036 (4.29)	0.870	211.6	0.035
Acute Care SMR-30 validation, Q1	92338	4038 (4.37)	0.871	39.1	0.035
Acute Care SMR-30 validation, Q2	94640	4258 (4.50)	0.869	49.1	0.037
Acute Care SMR-30 validation, Q3	95269	4023 (4.22)	0.867	66.3	0.034
Acute Care SMR-30 validation, Q4	91544	3717 (4.06)	0.873	88.4	0.033
ICU SMR-30 derivation	151426	13635 (9.00)	0.871	155.3	0.063
ICU SMR-30 validation	72160	6638 (9.20)	0.864	110.6	0.066
ICU SMR-30 validation, ICU level 1/2	62806	5746 (9.15)	0.866	103.0	0.065
ICU SMR-30 validation, ICU level 3/4	9354	892 (9.54)	0.848	18.0	0.071
ICU SMR-30 validation, Q1	17981	1652 (9.19)	0.864	29.3	0.066
ICU SMR-30 validation, Q2	18704	1787 (9.55)	0.863	32.1	0.069
ICU SMR-30 validation, Q3	18276	1681 (9.20)	0.865	36.3	0.066
ICU SMR-30 validation, Q4	17199	1518 (8.83)	0.862	31.8	0.064
Acute Care SMR derivation	853194	15429 (1.81)	0.916	247.6	0.015
Acute Care SMR validation	413329	7173 (1.74)	0.914	218.6	0.015
Acute Care SMR validation, Q1	102049	1802 (1.77)	0.918	57.8	0.015
Acute Care SMR validation, Q2	104446	1960 (1.88)	0.911	35.4	0.016
Acute Care SMR validation, Q3	105513	1759 (1.67)	0.912	75.0	0.014
Acute Care SMR validation, Q4	101321	1652 (1.63)	0.914	77.6	0.014
ICU SMR derivation	152914	9641 (6.30)	0.895	229.7	0.046
ICU SMR validation	72752	4555 (6.26)	0.887	153.0	0.047
ICU SMR validation, ICU level 1/2	63873	4122 (6.45)	0.887	128.0	0.048
ICU SMR validation, ICU level 3/4	8879	433 (4.88)	0.887	41.7	0.039
ICU SMR validation, Q1	18125	1138 (6.28)	0.887	23.8	0.047
ICU SMR validation, Q2	18881	1259 (6.67)	0.886	49.4	0.050
ICU SMR validation, Q3	18451	1145 (6.21)	0.888	42.0	0.046
ICU SMR validation, Q4	17295	1013 (5.86)	0.887	61.3	0.045

*ICU*, intensive care unit; *SMR*, standardized mortality ratio; *Q*, quarter; *C stats*, C statistic; *H-L GOF chi-square*, Hosmer Lemeshow goodness-of-fit C statistic chi-square value where a lower value is better and the size of the value is linearly related to the size of the cohort; all HL GOF chi-square values were significantly different (*p*<0.05). Level 1/2, ICUs who can provide most subspecialty medical and surgical Care; level 3/4: ICUs in smaller hospitals which lack some or many medical and surgical subspecialty care

**Figure 2:**
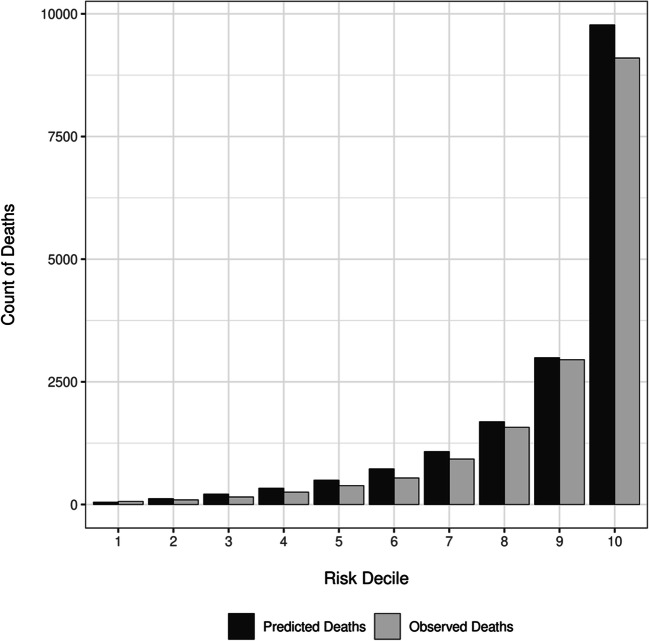
**Observed vs predicted mortality in the SMR-30 Validation Cohort using 10 equally sized bins defined by decile of predicted risk. This figure depicts the number of predicted and observed deaths in the validation cohort, stratified by decile of predicted risk for mortality. The number of hospitalizations per decile, as well observed and predicted number and proportion of deaths by decile are presented in Table** **3**.

The calibration plot (Fig. 2) and Table 3 show that the acute care SMR-30 model was well-calibrated in the validation cohort. There were 16,036 deaths (4.29% mortality) in the SMR-30 validation cohort versus 17,458 predicted deaths (4.67%), reflecting 0.38% over-prediction. Across deciles of predicted risk, the absolute difference in observed versus predicted percent mortality was a mean of 0.38%, with a maximum error of 1.81% seen in the highest-risk decile. Calibration plots and tables for the acute care SMR, ICU SMR, and ICU SMR-30 models are presented in Supplemental Figures 1–3 and Supplemental Tables 7–9. Similar to the acute care SMR-30 model, observed versus predicted mortality was within 1.0% for the acute care SMR, ICU SMR, and ICU SMR-30 validation cohorts. Additionally, mean error across risk deciles was <1.0%, and error greater than 1.0% was seen in only the highest risk decile of each model.

**Table 3:** Observed vs Predicted Mortality in the SMR-30 Validation Cohort Using 10 Equally Size Bins Defined by Decile of Predicted Risk

Risk decile	Predicted risk (lowest, highest)	Hospitalizations, *N*	Observed deaths, *N* (%)	Predicted deaths, *N* (%)	Difference*, *N* (%)
1	(0.000–0.002)	37,379	62 (0.17)	47 (0.13)	15 (0.04)
2	(0.002–0.004)	37,379	94 (0.25)	118 (0.32)	−24 (−0.06)
3	(0.004–0.007)	37,379	154 (0.41)	211 (0.56)	−57 (−0.15)
4	(0.007–0.011)	37,379	252 (0.67)	332 (0.89)	−80 (−0.21)
5	(0.011–0.016)	37,379	384 (1.03)	495 (1.33)	−111 (−0.30)
6	(0.016–0.023)	37,380	541 (1.45)	725 (1.94)	−184 (−0.49)
7	(0.023–0.035)	37,379	926 (2.48)	1077 (2.88)	−151 (−0.40)
8	(0.035–0.057)	37,379	1572 (4.21)	1686 (4.51)	−114 (−0.30)
9	(0.057–0.114)	37,379	2951 (7.89)	2991 (8.00)	−40 (−0.11)
10	(0.114–0.987)	37,379	9100 (24.35)	9775 (26.15)	−675 (−1.81)
1–10	(0.000–0.987)	373,791	16036 (4.29)	17458 (4.67)	−1422 (−0.38)

*Differences reflect the observed minus predicted mortality. Negative values indicate that the model over-predicted mortality, while positive value indicate that the model under-predicted mortality

The expected (mean) absolute calibration error^14^ across risk decile was 0.38%, while the maximum calibration error^14^ (observed in the highest risk decile) was 1.81%

## DISCUSSION

The VA was among the first healthcare systems to measure and report risk-adjusted ICU mortality. And, over the past 15 years, the VA’s mortality model has been updated, re-calibrated annually, and adapted for risk-adjustment of all VA acute care hospitalizations. In this study, we show that the VA’s mortality models (acute care SMR-30, acute care SMR, ICU SMR-30, and ICU SMR) can strongly discriminate in-hospital and 30-day mortality. Furthermore, the models are well-calibrated, with observed versus predicted mortality within 1% for all but the highest risk decile. Overall, the performance of each of the four VA mortality models is similar to the initial VA ICU mortality model,^11,19^ similar to other physiology-based mortality models such as APACHE, ^4,7,20,21^ and superior to risk models using administrative data only^22,23^. Likewise, the relatively lower calibration for the top risk-decile is consistent with other physiologic risk-adjustment models.^7^

A second major finding of our study is that the rates of inpatient and 30-day mortality for eligible acute care hospitalizations are relatively low (1.8% and 4.3%), which limits the ability to differentiate hospitals statistically based on mortality.^24^ Nonetheless, mortality monitoring is a critical component of quality measurement given the importance of identifying any hospitals with statistically greater-than-predicted mortality, as well as identifying numeric differences that may trigger further review to identify and remediate any problems before statistically significant differences in mortality arise. The strong performance of the VA mortality models lends credibility to their use in hospital evaluation and their ability to account for differences in patient case-mix across hospitals. However, mortality does not equate to quality. Greater-than-predicted mortality may occur for a number of reasons, not all of which reflect poor care. Thus, these mortality models serve as a warning tool to trigger deeper review, but are not a stand-alone marker of hospital quality. The results must be contextualized and evaluated alongside other metrics.

Several aspects of the modeling approach warrant further discussion. First, hospitalizations were assigned to one of 51 mutually exclusive admission diagnosis categories based on their admitting diagnosis, similar to the approach taken in the Kaiser Permanente Northern California’s risk-adjustment model^7^. By contrast, other models have used hierarchical approaches to classifying admission diagnoses, which are not based on diagnostic codes and must therefore be incorporated into workflow. For example, the UK’s Intensive Care National Audit and Research Centre’s coding method classifies ICU admissions by type (surgical, medical), system (e.g., respiratory), site (e.g., lungs), process (e.g., infection), and condition (e.g., bacterial pneumonia)^25^. While there are 741 unique conditions in this approach, five conditions accounted for 19.4% of all admissions,^25^ and the majority of unique conditions were ultimately excluded from the model to due imprecision in estimating the association between the condition and mortality (in which case hospitalizations are classified by the body system).^21^ The VA admission diagnosis categories each include one or more clinical classification software^8^(CCS) diagnosis categories. The merging of CCS categories into admission diagnosis categories was informed by clinical rationale, as well as by the observed mortality rates for CCS categories. For this reason, for example, upper and lower extremity fractures were merged together, while hip fracture was kept as a separate diagnosis category due to its higher associated mortality. The mapping of individual admission diagnoses to admission diagnosis categories via the CCS categories facilitates assignment of any new ICD-10-CM codes to an admission diagnosis category since the Agency for Healthcare Research and Quality updates the clinical classification software on an ongoing basis. While some clinicians may prefer more granular admission diagnosis groupings, each group must have a sufficient number of observations to estimate the association with mortality—limiting the number of discrete diagnosis groupings that can be used in practice. Instead, the physiologic variables serve to further differentiate the hospitalizations within the same diagnosis category, and consistently provide far more prognostic information than the diagnosis category.^7,11^

Second, hospitalizations were excluded from the VA model if the patient had a hospice encounter in the year preceding or on the calendar day of admission. Only 1.5% and 0.1% of otherwise eligible hospitalizations were excluded, respectively, due to hospice encounters prior to or on the day of admission. Furthermore, in exploratory analyses without this exclusion, the mortality models perform similarly since the model consistently identifies patients referred to hospice as having a high risk for mortality. Some clinicians may argue for expanding the hospice exclusion to also exclude patients with who transition to hospice at later points in hospitalization. However, a majority of patients who die during inpatient hospitalization are transitioned to comfort-only measures or have treatment limitations initiated prior to death, such that broad exclusions of patients with hospice care could substantially limit the ability to differentiate mortality outcomes across hospitals. Initiation of hospice care during or before the calendar day of admission was felt to be the fairest approach. However, the best approach to incorporating treatment limitations into hospital performance assessment remains an area of ongoing study, and best practices are yet to be defined.^26^ Through the VA’s Life-Sustaining Treatment Decisions Initiative, there is a national effort to elicit, honor, and document Veteran’s values, goals, and healthcare treatment preferences. The initiative’s harmonized approach to documenting treatment preferences across VA hospitals may allow for future incorporation of treatment preferences documented at hospital admission to be incorporated into performance measurement.

Third, physiological variables are currently incorporated into the VA’s mortality models as categorical variables, which allow for ready interpretation of the association between physiologic derangements and the risk of mortality. By contrast, some other models (and VA’s initial ICU mortality model) use cubic splines^3,7,11^—which allow for more flexible parameterization of the physiologic variables, but come at the cost of decreased transparency, since the model output is not readily interpretable. The opaqueness of regression models has been cited as a key drawback of regression-based performance assessment, which may reduce credibility and motivation to act on the assessment results.^27^ Thus, given the trade-offs between statistical precision and interpretability, there is no “best approach” to the incorporation of physiologic variables. The current VA mortality models using categorical physiologic variables perform similarly to the prior VA ICU mortality model using cubic splines, indicating that the loss of performance is minimal, and therefore, the added statistical precision may not be worth the added complexity of interpretation.

There are some limitations to acknowledge. First, there are many drawbacks to the use of risk-adjusted mortality for measuring hospital quality, which are discussed in detail elsewhere, including low power, inability to differentiate preventable versus unpreventable deaths, and the imperfect correlation between process and outcome measures^28,29^. Despite these limitations, monitoring risk-adjusted mortality is an important component of quality improvement, as discussed above. Secondly, the VA’s acute care mortality models incorporate 8 physiologic variables (sodium, BUN, creatinine, glucose, albumin, bilirubin, white blood cell count, and hematocrit), with an additional three values (PaO_2_, PaCO_2_, and pH) included in the ICU models. These physiologic variables are commonly included in other physiologic risk-adjustment models and have high clinical face value, but are not fully comprehensive. Additional physiologic measurements such as vital signs (heart rate, blood pressure, respiratory rate, pulse oximetry), mental status, and blood lactate measurement may provide additional prognostic information^30^. Vital signs and mental status cannot be readily incorporated into the VA’s mortality model at present because they are recorded outside the electronic health record (e.g., in ICU-specific programs) in many units, leading to systematic missingness that could bias risk adjustment. Lactate measurements, however, are available in the electronic health record, and are currently being considered for incorporation into VA mortality models. Finally, the VA patient population has unique demographics, risk factors, and comorbidity profile, so this model may not generalize to other settings. Indeed, model performance often degrades when applying models to new settings, underscoring the need for periodic model evaluation and recalibration and the benefit of developing context-specific models rather than simply applying “off-the-shelf” risk tools^5,21^.

## CONCLUSIONS

We have shown that the VA’s mortality models, which incorporate patient physiology and are recalibrated annually using hospitalizations from the prior 2 years, are highly predictive and have good calibration both overall and across risk deciles. The strong model performance underscores the benefit of physiologic data and the development of models in the population and setting in which they will be used.

## Supplementary Information

ESM 1 (DOCX 176 kb)

## References

[1] Fihn SD, Francis J, Clancy C (2014). Insights from advanced analytics at the Veterans Health Administration. Health Aff (Millwood)..

[2] Render ML, Kim HM, Welsh DE (2003). Automated intensive care unit risk adjustment: results from a National Veterans Affairs study. Crit Care Med..

[3] Render ML, Deddens J, Freyberg R (2008). Veterans Affairs intensive care unit risk adjustment model: validation, updating, recalibration. Crit Care Med..

[4] **Zimmerman JE, Kramer AA, McNair DS, Malila FM.** Acute Physiology and Chronic Health Evaluation (APACHE) IV: hospital mortality assessment for today’s critically ill patients. *Crit Care Med.* 2006;34:1297-1310. 10.1097/01.CCM.0000215112.84523.F0 16540951

[5] Harrison DA, Brady AR, Parry GJ, Carpenter JR, Rowan K (2006). Recalibration of risk prediction models in a large multicenter cohort of admissions to adult, general critical care units in the United Kingdom. Crit Care Med..

[6] Steyerberg EW, Vickers AJ, Cook NR (2010). Assessing the performance of prediction models: a framework for traditional and novel measures. Epidemiology..

[7] Escobar GJ, Greene JD, Scheirer P, Gardner MN, Draper D, Kipnis P (2008). Risk-adjusting hospital inpatient mortality using automated inpatient, outpatient, and laboratory databases. Med Care..

[8] HCUP. Beta Clinical Classifications Software (CCS) for ICD-10-CM/PCS. https://www.hcup-us.ahrq.gov/toolssoftware/ccs10/ccs10.jsp. Accessed September 11, 2019.

[9] Elixhauser A, Steiner C, Harris DR, Coffey RM (1998). Comorbidity measures for use with administrative data. Med Care..

[10] Healthcare Cost and Utilization Project. Elixhauser Comorbity Software Redefined for ICD-10-CM. Available at https://www.hcup-us.ahrq.gov/toolssoftware/comorbidityicd10/comorbidity_icd10.jsp (accessed May 2, 2021). Accessed.

[11] Render ML, Kim HM, Welsh DE (2003). Automated intensive care unit risk adjustment: results from a National Veterans Affairs study. Critical Care Medicine..

[12] Altman DG, Vergouwe Y, Royston P, Moons KG (2009). Prognosis and prognostic research: validating a prognostic model. BMJ..

[13] Royston P, Moons KG, Altman DG, Vergouwe Y (2009). Prognosis and prognostic research: Developing a prognostic model. BMJ..

[14] Huang Y, Li W, Macheret F, Gabriel RA, Ohno-Machado L (2020). A tutorial on calibration measurements and calibration models for clinical prediction models. J Am Med Inform Assoc..

[15] **Hosmer DW, Lemeshow S.** Applied logistic regression. *Wiley series in probability and mathematical statistics Applied probability and statistics.* 1989.

[16] **Hosmer DW, Lemeshow S.** Applied logistic regression. *Wiley series in probability and statistics Texts and references section.* 2000.

[17] Hilbert G, Gruson D, Vargas F (2001). Noninvasive ventilation in immunosuppressed patients with pulmonary infiltrates, fever, and acute respiratory failure. N Engl J Med..

[18] Almenoff P, Sales A, Rounds S (2007). Intensive care services in the Veterans Health Administration. Chest..

[19] **Elder NC, Brungs SM, Nagy M, Kudel I, Render ML.** Intensive care unit nurses’ perceptions of safety after a highly specific safety intervention. *Qual Saf Health Care.* 2008;17:25-30. 10.1136/qshc.2006.021949 18245216

[20] Liu V, Turk BJ, Ragins AI, Kipnis P, Escobar GJ (2013). An electronic Simplified Acute Physiology Score-based risk adjustment score for critical illness in an integrated healthcare system. Critical Care Medicine..

[21] Harrison DA, Parry GJ, Carpenter JR, Short A, Rowan K (2007). A new risk prediction model for critical care: the Intensive Care National Audit & Research Centre (ICNARC) model. Crit Care Med..

[22] Yale New Haven Health System/ Center for Outcomes Research & Evaluation (YNHHS/CORE). Hospital-Wide (All-Condition, All-Procedure) Risk-Standardized Mortality Measure: Draft Measure Methodology for Interim Public Comment. https://www.cms.gov/Medicare/Quality-Initiatives-Patient-Assessment-Instruments/MMS/Downloads/Hospital-Wide_All-Condition_All-Procedure_Risk-Standardized-Mortality-Measure_Public-Comment.pdf. Published 2016. Accessed November 24, 2021.

[23] Krumholz HM, Coppi AC, Warner F (2019). Comparative Effectiveness of New Approaches to Improve Mortality Risk Models From Medicare Claims Data. JAMA Netw Open..

[24] Krell RW, Hozain A, Kao LS, Dimick JB (2014). Reliability of risk-adjusted outcomes for profiling hospital surgical quality. JAMA Surg..

[25] Young JD, Goldfrad C, Rowan K (2001). Development and testing of a hierarchical method to code the reason for admission to intensive care units: the ICNARC Coding Method. Intensive Care National Audit & Research Centre. Br J Anaesth..

[26] Walkey AJ, Weinberg J, Wiener RS, Cooke CR, Lindenauer PK (2016). Association of Do-Not-Resuscitate Orders and Hospital Mortality Rate Among Patients With Pneumonia. JAMA Intern Med..

[27] **Pronovost PJ, Austin JM, Cassel CK, et al.** Fostering Transparency in Outcomes, Quality, Safety, and Costs: A Vital Direction for Health and Health Care | National Academy of Medicine. 2016. 10.1001/jama.2016.14039 27669332

[28] **Lilford R, Pronovost P.** Using hospital mortality rates to judge hospital performance: a bad idea that just won’t go away. *BMJ.* 2010;340:c2016. 10.1136/bmj.c2016 20406861

[29] Holloway RG, Quill TE (2007). Mortality as a measure of quality: implications for palliative and end-of-life care. JAMA..

[30] Escobar GJ, Gardner MN, Greene JD, Draper D, Kipnis P (2013). Risk-adjusting hospital mortality using a comprehensive electronic record in an integrated health care delivery system. Medical Care..

