# Supplementary material for: Risk-Adjusting Mortality in the Nationwide Veterans Affairs Healthcare System
Source: J Gen Intern Med. 2022 Jan 13;37(15):3877–84. doi: 10.1007/s11606-021-07377-1 (PMC9640507; doi:10.1007/s11606-021-07377-1)
Supplement: Supplementary file 1 — (DOCX 176 kb) [file 11606_2021_7377_MOESM1_ESM.docx]

**ONLINE SUPPLEMENT** to Risk-Adjusting Mortality in the Nationwide Veterans Affairs Healthcare System

**TABLE OF CONTENTS**

**Appendix 1**: Description of 4 standardized mortality ratios (SMRs) Page 2

**Supplemental Table 1**: Key differences between the SMR models Page 6

**Supplemental Table 2:** Recent Updates to the VA’s SMR models Page 7

**Supplemental Table 3**: Study Flow Diagram for SMR-30 models Page 8

**Supplemental Table 4**: Study Flow Diagram for SMR models Page 8

**Supplemental Table 5**: Unique patients in Acute Care and ICU SMR models Page 8

**Supplemental Table 6**: C-statistic for Nested Acute Care SMR-30 Models Page 9

**Supplemental Figure 1**: Calibration Plot for acute care SMR Page 10

**Supplemental Table 7**: Observed vs predicted mortality,

Acute Care SMR Validation Page 10

**Supplemental Figure 2**: Calibration Plot for ICU SMR-30 Page 11

**Supplemental Table 8**: Observed vs predicted mortality,

ICU SMR-30 Validation Page 11

**Supplemental Figure 3**: Calibration Plot for ICU SMR Page 12

**Supplemental Table 9**: Observed vs predicted mortality,

ICU SMR Validation Page 12

**Appendix 1**: Description of 4 standardized mortality ratios (SMRs)

| **1. Acute Care Hospital Standardized Mortality Ratio (SMR)** | |
| --- | --- |
| Measure Description | The number of observed deaths that occur compared to the number of deaths predicted to occur during hospitalization or within one calendar day of hospital discharge at a location. |
| Cohort Definition | The first acute care (including ICU) hospital admission at a location, based on physician treating specialties, with a discharge date in the time frame being reported. |
| Cohort Exclusions | Patients who have died within 4 hours of hospital admission.  Patients with a hospice care one year prior to or calendar day of hospital admission. Hospice care is identified as any of the following circumstances one year prior to or calendar day of hospital admission: Inpatient Treating Specialty of Hospice; Outpatient Hospice encounter; Discharge Disposition of Hospice; Hospice claims; or Hospice CPT codes  Transplant patients who receive their transplantation within the current hospitalization.  Nursing home, psychiatry, rehabilitation, observation, and hospice physician treating specialties.  Patients whose discharge status was TRANSFER.  Patients who received an LVAD (left ventricle assist device) one year prior to or during hospitalization. |
| Numerator Definition | The number of patients with an acute care (including ICU) hospital admission that died during hospitalization or within one calendar day of hospital discharge in the time frame being reported. |
| Denominator Definition | The number of deaths predicted to occur during hospitalization or within one calendar day of hospital discharge based on the individual patients' predicted mortality in the time frame being reported. |
| Predicted Deaths | Predicted mortality is estimated using a logistic regression model that has as predictors: age, marital status, admitting diagnosis category (51 mutually exclusive grouping based on consolidated clinical classification software groupings), major surgical procedure within 24 hours of presentation, 29 comorbid conditions, physiologic variables (Sodium, BUN, Creatinine, Glucose, Albumin, Bilirubin, WBC, HCT), immunosuppressant status, ICU stay during hospitalization, medical or surgical DRG, and source of admission. The most deranged lab value within a specified time frame is included in this statistical model. For non-operative patients, this time frame is between 24 hours prior to hospital admission and 24 hours after hospital admission. For operative patients, this time frame is between 14 days prior to hospital admission and 24 hours after hospital admission. |

| **2. Acute Care 30-Day Standardized Mortality Ratio (SMR-30)** | |
| --- | --- |
| Measure Description | The number of observed deaths that occur within 30 days of hospital admission compared to the number of deaths predicted to occur within 30 days of hospital admission at a location. |
| Cohort Definition | The first acute care (including ICU) hospital admission at a location, based on physician treating specialties, with a discharge date in the time frame being reported. |
| Cohort Exclusions | Patients who have died within 4 hours of hospital admission.  Patients with a hospice care one year prior to or calendar day of hospital admission. Hospice care is identified as any of the following circumstances one year prior to or calendar day of hospital admission: Inpatient Treating Specialty of Hospice; Outpatient Hospice encounter; Discharge Disposition of Hospice; Hospice claims; or Hospice CPT codes  Transplant patients who receive their transplantation within the current hospitalization.  Nursing home, psychiatry, rehabilitation, observation, and hospice physician treating specialties.  Multiple hospital admissions within 30 days of an index admission.  Patients who received an LVAD (left ventricle assist device) one year prior to or during hospitalization. |
| Numerator Definition | The number of patients with an acute care (including ICU) hospital admission that died during hospitalization or within one calendar day of hospital discharge in the time frame being reported. |
| Denominator Definition | The number of deaths predicted to occur during hospitalization or within one calendar day of hospital discharge based on the individual patients' predicted mortality in the time frame being reported. |
| Predicted Deaths | Predicted mortality is estimated using a logistic regression model that has as predictors: age, marital status, admitting diagnosis category, major surgical procedure within 24 hours of presentation, 29 comorbid conditions, physiologic variables (Sodium, BUN, Creatinine, Glucose, Albumin, Bilirubin, WBC, HCT), immunosuppressant status, ICU stay during hospitalization, medical or surgical DRG, and source of admission. The most deranged lab value within a specified time frame is included in this statistical model. For non-operative patients, this time frame is between 24 hours prior to hospital admission and 24 hours after hospital admission. For operative patients, this time frame is between 14 days prior to hospital admission and 24 hours after hospital admission. |

| **3. ICU Hospital Standardized Mortality Ratio (SMR)** | |
| --- | --- |
| Measure Description | The number of observed deaths that occur compared to the number of deaths predicted to occur during hospitalization or within one calendar day of hospital discharge at a location. |
| Cohort Definition | The first ICU hospital admission at a location, based on physician treating specialties, with a discharge date in the time frame being reported. |
| Cohort Exclusions | Patients who have died within 4 hours of hospital admission.  Patients with a hospice care one year prior to or calendar day of hospital admission. Hospice care is identified as any of the following circumstances one year prior to or calendar day of hospital admission: Inpatient Treating Specialty of Hospice; Outpatient Hospice encounter; Discharge Disposition of Hospice; Hospice claims; or Hospice CPT codes  Transplant patients who receive their transplantation within the current hospitalization.  Nursing home, psychiatry, rehabilitation, observation, and hospice physician treating specialties.  Patients whose discharge status was TRANSFER.  Patients who received an LVAD (left ventricle assist device) one year prior to or during hospitalization. |
| Numerator Definition | The number of patients with an ICU hospital admission at a location based on physician treating specialty, that died during hospitalization or within one calendar day of hospital discharge in the time frame being reported. |
| Denominator Definition | The number of deaths with an ICU hospital admission by physician treating specialty, predicted to occur during hospitalization or within one calendar day of hospital discharge based on the individual patients' predicted mortality in the time frame being reported. |
| Predicted Deaths | Predicted mortality is estimated using a logistic regression model that has as predictors: age, marital status, diagnosis/procedure, 29 comorbid conditions, physiologic variables (Sodium, BUN, Creatinine, Glucose, Albumin, Bilirubin, WBC, HCT and arterial blood gases (PAO2, PCO2 and Ph)), immunosuppressant status, length of stay in hospital prior to ICU admission, medical or surgical DRG, and source of admission. The most deranged lab value within a specified time frame is included in this statistical model. For non-operative patients, this time frame is between 24 hours prior to ICU admission and 24 hours after ICU admission. For operative patients, this time frame is between 14 days prior to ICU admission and 24 hours after ICU admission. |

| **4. ICU 30-Day Standardized Mortality Ratio (SMR-30)** | |
| --- | --- |
| Measure Description | The number of observed deaths that occur within 30 days of hospital admission compared to the number of deaths predicted to occur within 30 days of hospital admission at a location. |
| Cohort Definition | The first ICU hospital admission at a location, based on physician treating specialties, with a discharge date in the time frame being reported. |
| Cohort Exclusions | Patients who have died within 4 hours of hospital admission.  Patients with a hospice care one year prior to or calendar day of hospital admission. Hospice care is identified as any of the following circumstances one year prior to or calendar day of hospital admission: Inpatient Treating Specialty of Hospice; Outpatient Hospice encounter; Discharge Disposition of Hospice; Hospice claims; or Hospice CPT codes  Transplant patients who receive their transplantation within the current hospitalization.  Nursing home, psychiatry, rehabilitation, observation, and hospice physician treating specialties.  Multiple hospital admissions within 30 days of an index admission.  Patients who received an LVAD (left ventricle assist device) one year prior to or during hospitalization. |
| Numerator Definition | The number of patients with an ICU hospital admission at a location based on physician treating specialty, that died during hospitalization or within one calendar day of hospital discharge in the time frame being reported. |
| Denominator Definition | The number of deaths with an ICU hospital admission by physician treating specialty, predicted to occur during hospitalization or within one calendar day of hospital discharge based on the individual patients' predicted mortality in the time frame being reported. |
| Predicted Deaths | Predicted mortality is estimated using a logistic regression model that has as predictors: age, marital status, diagnosis/procedure, 29 comorbid conditions, physiologic variables (Sodium, BUN, Creatinine, Glucose, Albumin, Bilirubin, WBC, HCT and arterial blood gases (PAO2, PCO2 and Ph)), immunosuppressant status, length of stay in hospital prior to ICU admission, medical or surgical DRG, and source of admission. The most deranged lab value within a specified time frame is included in this statistical model. For non-operative patients, this time frame is between 24 hours prior to ICU admission and 24 hours after ICU admission. For operative patients, this time frame is between 14 days prior to ICU admission and 24 hours after ICU admission. |

| **Supplemental Table 1**: Key differences between the 4 SMR models | | | |
| --- | --- | --- | --- |
| Model | Admission Location | Discharged as a transfer | Multiple admissions within the reporting timeframe |
| Acute care SMR-30 | Any | Include | 1 per 30 days |
| Acute care SMR | Any | Exclude | All included |
| ICU SMR-30 | ICU only | Include | 1 per 30 days |
| ICU SMR | ICU only | Exclude | All included |

| **Supplemental Table 2:** Recent Updates to the VA’s SMR models | | |
| --- | --- | --- |
| Year | Update | Rationale |
| 2015-2016 | Diagnosis groupings updated to include ICD10 codes. | National conversion from ICD9 to ICD10 diagnosis coding. |
| 2018 | Hospice care definition updated to include CPT codes. | Allows more complete identification of hospice care. |
| 2018 | Updated process for identifying ICU patients based on treating specialty rather than bed location. | Allows more accurate identification of ICU level of care. |
| 2018 | Laboratory values converted from continuous splined variables to categorical variables. | Allows for more intuitive interpretation of model coefficients. |
| 2019 | Converted from original diagnoses groupings to new diagnosis groupings based on aggregated HCUP CCSR groups. | The diagnosis groupings facilitate the assignment of any new ICD10 codes to a diagnosis group (via the HCUP CCSR group). |
| Annually | Diagnoses of AMI, COPD, pneumonia, CHF, and stroke updated to align with CMS definitions. | Ensures harmonization with CMS definitions. |

| **Supplemental Table 3**: Study Flow Diagram for SMR-30 Models. | | |
| --- | --- | --- |
|  | Acute Care  SMR-30  Model | ICU  SMR-30 Model |
| Total Inpatient Stays FY 2017-2019 | 1,996,645 | 239,661 |
| Treating specialty is not nursing home, psychiatry, rehabilitation, observation, or hospice | 1,322,832 | 239,661 |
| Hospitalization within 30 days of a prior hospitalization during the same quarter | 1,208,764 | 229,235 |
| No transplant | 1,207,911 | 228,583 |
| No LVAD in the year prior to or during hospitalization | 1,207,484 | 228,351 |
| Survived more than 4 hours from admission | 1,207,069 | 228,035 |
| No hospice event in the 1 year prior to admission | 1,188,507 | 224,113 |
| No hospice event on calendar day of admission | 1,187,058 | 223,795 |

| **Supplemental Table 4**: Study Flow Diagram for SMR Models. | | |
| --- | --- | --- |
|  | Acute Care  SMR Model | ICU  SMR Model |
| Total Hospitalizations FY 2017-2019 | 1,996,645 | 239,661 |
| Treating specialty is not nursing home, psychiatry, rehabilitation, observation, or hospice | 1,332,832 | 239,661 |
| No transplant | 1,322,141 | 238,985 |
| Discharge status is not a transfer | 1,292,159 | 230,822 |
| No LVAD in the prior to or during hospitalization | 1,291,704 | 230,566 |
| Survived more that 4 hours from admission | 1,291,233 | 230,248 |
| No hospice event in the 1 year prior to admission | 1,268,617 | 226,061 |
| No hospice event on the calendar day of admission | 1,266,903 | 225,726 |

| **Supplemental Table 5**: Unique patients in Acute Care and ICU SMR models | | | |
| --- | --- | --- | --- |
|  | Hospitalizations, N | Unique Patients, N | Hospitalizations per Patient, Mean |
| Acute Care SMR derivation | 853,194 | 513,428 | 1.66 |
| Acute Care SMR validation, full year | 413,329 | 285,801 | 1.45 |
| Acute Care SMR validation, Q1 | 102,049 | 86,476 | 1.18 |
| Acute Care SMR validation, Q2 | 104,446 | 88,944 | 1.17 |
| Acute Care SMR validation, Q3 | 105,513 | 89,422 | 1.18 |
| Acute Care SMR validation, Q4 | 101,321 | 85,667 | 1.18 |
| ICU SMR derivation | 152,914 | 127,885 | 1.20 |
| ICU SMR validation | 72,752 | 64,014 | 1.14 |
| ICU SMR validation, Q1 | 18,125 | 17,182 | 1.05 |
| ICU SMR validation, Q2 | 18,881 | 17,913 | 1.05 |
| ICU SMR validation, Q3 | 18,451 | 17,530 | 1.05 |
| ICU SMR validation, Q4 | 17,295 | 16,415 | 1.05 |

| **Supplemental Table 6**: C-statistic for Nested Acute Care SMR-30 Models | | |
| --- | --- | --- |
|  | Derivation (N=769,710) | Validation (N=373,791) |
| Comprehensive Clinical | 0.871 | 0.870 |
| Enhanced Administrative | 0.854 | 0.853 |
| Basic Administrative | 0.842 | 0.840 |
| The basic administrative model includes age, comorbidity, admission type, diagnosis, and procedure data. The enhanced administrative model adds marital status, admission source, and ICU use. The comprehensive clinical model adds immunosuppressed status and admission laboratory data. | | |

**Supplemental Figure 1**: Calibration Plot for **acute care SMR Validation** using 10 equally-size bins defiled by decile of predicted risk


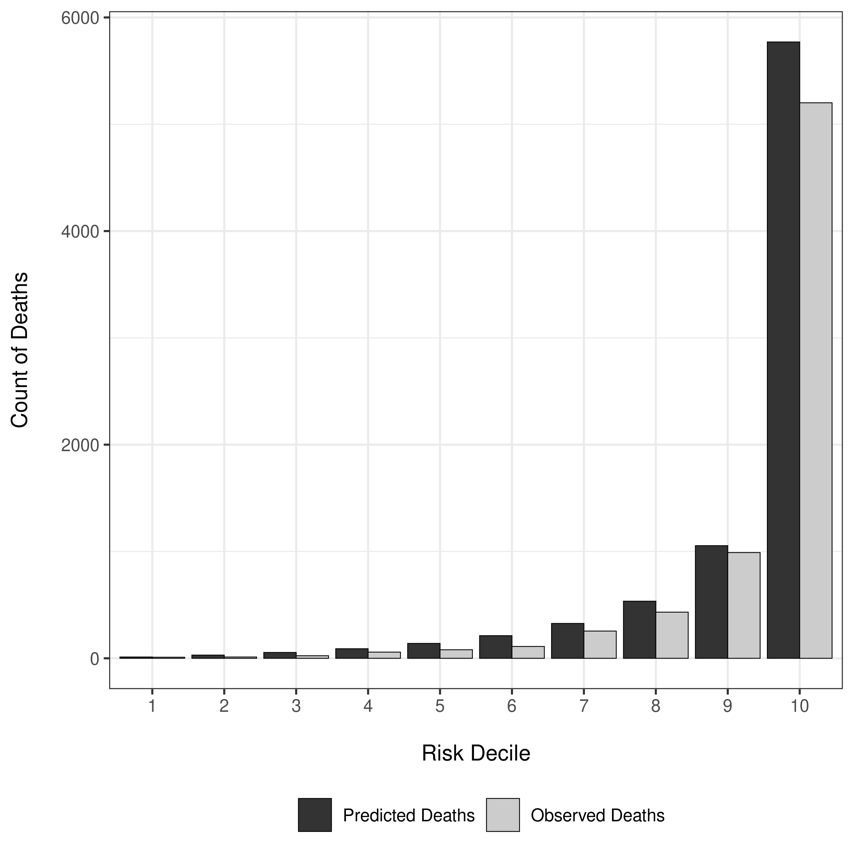


| **Supplemental Table 7**: Observed vs predicted mortality in the **acute care SMR Validation Cohort** using 10 equally-size bins defined by decile of predicted risk | | | | | |
| --- | --- | --- | --- | --- | --- |
| Risk Decile | Predicted risk (lowest, highest) | Hospitalizations, N | Observed Deaths, N (%) | Predicted Deaths, N (%) | Difference*, N (%) |
| 1 | (0.000-0.000) | 41332 | 10 (0.02) | 12 (0.03) | -2 (-0.00) |
| 2 | (0.000-0.001) | 41333 | 12 (0.03) | 30 (0.07) | -18 (-0.04) |
| 3 | (0.001-0.002) | 41333 | 24 (0.06) | 55 (0.13) | -31 (-0.08) |
| 4 | (0.002-0.003) | 41332 | 58 (0.14) | 90 (0.22) | -32 (-0.08) |
| 5 | (0.003-0.004) | 41334 | 80 (0.19) | 139 (0.34) | -59 (-0.14) |
| 6 | (0.004-0.006) | 41332 | 111 (0.27) | 212 (0.51) | -101 (-0.24) |
| 7 | (0.006-0.010) | 41334 | 255 (0.62) | 326 (0.79) | -71 (-0.17) |
| 8 | (0.010-0.017) | 41332 | 432 (1.05) | 535 (1.29) | -103 (-0.25) |
| 9 | (0.017-0.039) | 41334 | 990 (2.40) | 1055 (2.55) | -65 (-0.16) |
| 10 | (0.039-0.985) | 41333 | 5201 (12.58) | 5770 (13.96) | -569 (-1.38) |
| 1-10 | **(0.000-0.985)** | **413329** | **7173 (1.74)** | **8224 (1.99)** | **-1051 (-0.25)** |
| *Differences reflect the observed minus predicted mortality. Negative values indicate that the model over-predicted mortality, while positive value indicate that the model under-predicted mortality.  The mean absolute calibration error across risk deciles was 0.25%, while the maximum calibration error (observed in the highest risk decile) was 1.38%. | | | | | |

**Supplemental Figure 2**: Calibration Plot for **ICU SMR-30 Validation** using 10 equally-size bins defiled by decile of predicted risk


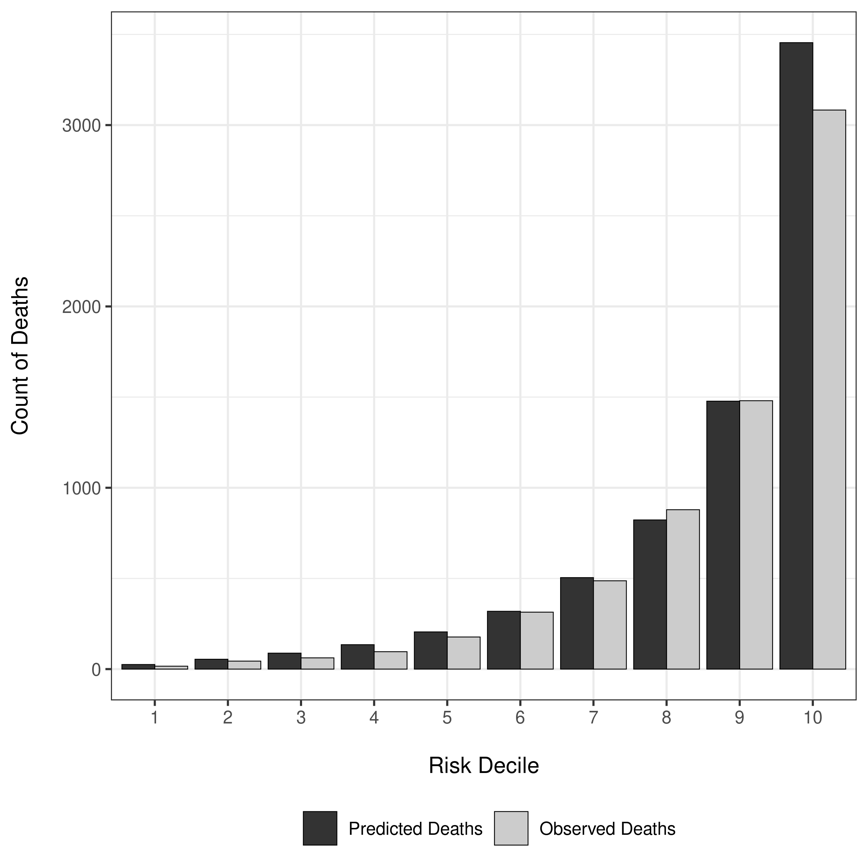


| **Supplemental Table 8**: Observed vs predicted mortality in the **ICU SMR-30 Validation Cohort** using 10 equally-size bins defined by decile of predicted risk | | | | | |
| --- | --- | --- | --- | --- | --- |
| Risk Decile | Predicted risk (lowest, highest) | Hospitalizations, N | Observed Deaths, N (%) | Predicted Deaths, N (%) | Difference*, N (%) |
| 1 | (0.000-0.006) | 7216 | 16 (0.22) | 25 (0.35) | -9 (-0.12) |
| 2 | (0.006-0.010) | 7216 | 44 (0.61) | 54 (0.75) | -10 (-0.14) |
| 3 | (0.010-0.015) | 7216 | 62 (0.86) | 88 (1.22) | -26 (-0.36) |
| 4 | (0.015-0.023) | 7216 | 96 (1.33) | 135 (1.87) | -39 (-0.54) |
| 5 | (0.023-0.035) | 7216 | 177 (2.45) | 205 (2.84) | -28 (-0.39) |
| 6 | (0.035-0.055) | 7216 | 314 (4.35) | 319 (4.42) | -5 (-0.07) |
| 7 | (0.055-0.088) | 7216 | 487 (6.75) | 505 (7.00) | -18 (-0.25) |
| 8 | (0.088-0.147) | 7216 | 879 (12.18) | 823 (11.41) | 56 (0.78) |
| 9 | (0.147-0.283) | 7216 | 1480 (20.51) | 1478 (20.48) | 2 (0.03) |
| 10 | (0.283-0.987) | 7216 | 3083 (42.72) | 3455 (47.88) | -372 (-5.16) |
| 1-10 | **(0.000-0.987)** | **72160** | **6638 (9.20)** | **7086 (9.82)** | **-448 (-0.62)** |
| *Differences reflect the observed minus predicted mortality. Negative values indicate that the model over-predicted mortality, while positive value indicate that the model under-predicted mortality.  The mean absolute calibration error across risk deciles was 0.62%, while the maximum calibration error (observed in the highest risk decile) was 5.16%. | | | | | |

**Supplemental Figure 3**: Calibration Plot for **ICU SMR Validation** using 10 equally-size bins defiled by decile of predicted risk


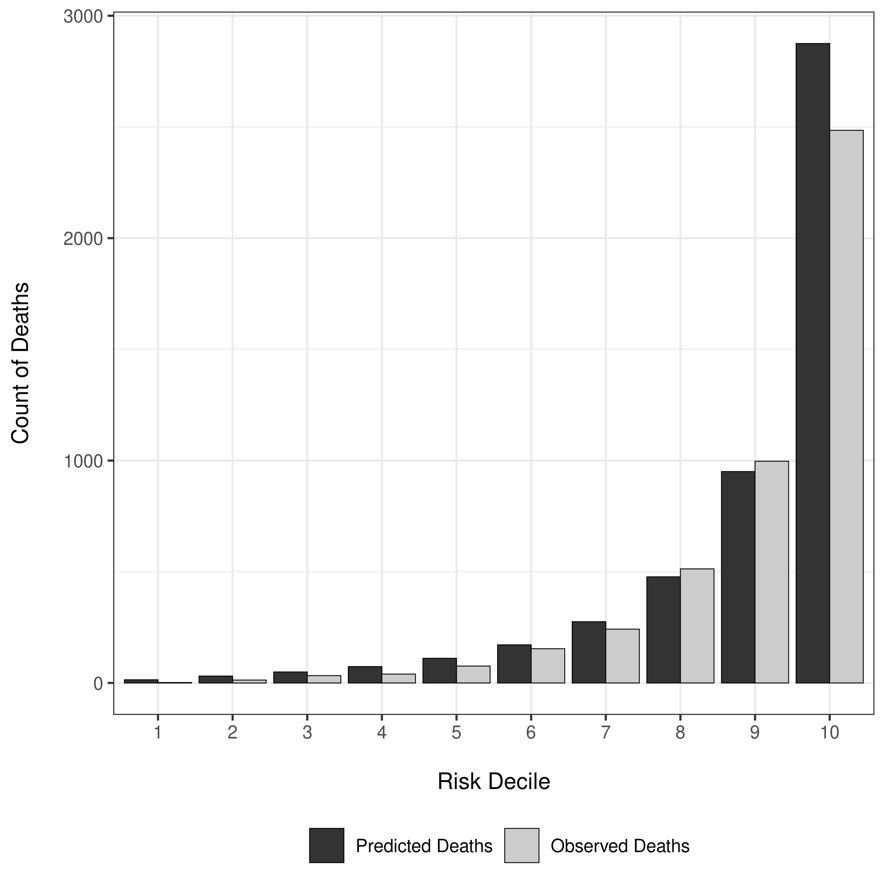


| **Supplemental Table 9**: Observed vs predicted mortality in the **ICU SMR Validation Cohort** using 10 equally-size bins defined by decile of predicted risk | | | | | |
| --- | --- | --- | --- | --- | --- |
| Risk Decile | Predicted risk (lowest, highest) | Hospitalizations, N | Observed Deaths, N (%) | Predicted Deaths, N (%) | Difference*, N (%) |
| 1 | (0.000-0.003) | 7275 | 2 (0.03) | 14 (0.20) | -12 (-0.20) |
| 2 | (0.003-0.005) | 7275 | 13 (0.18) | 31 (0.43) | -18 (-0.23) |
| 3 | (0.005-0.008) | 7275 | 33 (0.45) | 50 (0.68) | -17 (-0.18) |
| 4 | (0.008-0.012) | 7276 | 40 (0.55) | 74 (1.01) | -34 (-0.51) |
| 5 | (0.012-0.019) | 7275 | 76 (1.04) | 111 (1.53) | -35 (-0.53) |
| 6 | (0.019-0.029) | 7275 | 154 (2.12) | 171 (2.36) | -17 (-0.26) |
| 7 | (0.029-0.048) | 7276 | 242 (3.33) | 276 (3.79) | -34 (-0.49) |
| 8 | (0.048-0.088) | 7275 | 513 (7.05) | 477 (6.56) | 36 (0.54) |
| 9 | (0.088-0.194) | 7275 | 997 (13.70) | 951 (13.07) | 46 (0.63) |
| 10 | (0.194-0.991) | 7275 | 2485 (34.16) | 2875 (39.52) | -390 (-5.32) |
| 1-10 | **(0.000-0.991)** | **72752** | **4555 (6.26)** | **5030 (6.91)** | **-475 (-0.65)** |
| *Differences reflect the observed minus predicted mortality. Negative values indicate that the model over-predicted mortality, while positive value indicate that the model under-predicted mortality.  The mean absolute calibration error across risk deciles was 0.65%, while the maximum calibration error (observed in the highest risk decile) was 5.32%. | | | | | |
